# Potential Health Risk of Microplastic Exposures from Skin-Cleansing Products

**DOI:** 10.3390/toxics13050354

**Published:** 2025-04-29

**Authors:** Raluca Maria Bucur (Popa), Cristiana Radulescu, Ioana Daniela Dulama, Raluca Maria Stirbescu, Ioan Alin Bucurica, Andreea Laura Banica, Sorina Geanina Stanescu

**Affiliations:** 1 Doctoral School Chemical Engineering and Biotechnology, National University of Science and Technology Politehnica of Bucharest, 060042 Bucharest, Romania; ralucamaria.bucur@gmail.com (R.M.B.); banica.andreea@icstm.ro (A.L.B.); 2 Research and Expertise Center for Natural Resources and Environment, Valahia University of Targoviste, 130004 Targoviste, Romania; 3 Faculty of Sciences and Arts, Valahia University of Targoviste, 130004 Targoviste, Romania; 4 Academy of Romanian Scientists, 050044 Bucharest, Romania; 5 Institute of Multidisciplinary Research for Science and Technology, Valahia University of Targoviste, 130004 Targoviste, Romania; stirbescu.raluca@icstm.ro (R.M.S.); bucurica_alin@icstm.ro (I.A.B.); geanina.stanescu@icstm.ro (S.G.S.)

**Keywords:** skin cleansers, microplastics, optical microscopy, micro-FTIR, statistical approach, health risk, ingestion dose, inhalation dose, absorption dose, chronic total exposure

## Abstract

This research aims to investigate and quantify the possible presence of microplastics (MPs) in usual skin-cleansing products (i.e., liquid soap, micellar water, and micellar cleansing oil), the most popular from the market in terms of brand and customer confidence. Therefore, optical microscopy and micro-Fourier transform infrared spectroscopy (micro-FTIR) were used to determine the MPs’ number, color, shape, size, and chemical composition. For the first time, the results were correlated with the possible exposure paths (i.e., inhalation, ingestion, or adsorption) to assess the human health risk of the analyzed micellar-based cleansers in terms of chronic total exposure dose to microplastics. Finally, a statistical analysis was added to this study for source prediction of MPs in skin-cleansing samples in terms of morphology, chemical composition, and other factors (i.e., brand, packaging, etc.). The various exposures and toxicities of MPs were assessed in terms of potential health risk, knowing that their toxic effect depends on the polymeric structure strongly linked with the size, shape, and concentration in the products.

## 1. Introduction

The cosmetics sector represents a huge global market worth billions of dollars related to dermato-cosmetics products with antibacterial properties (mainly for skin care in terms of internal and external treatment). In this regard, the cosmetic sector and researchers in the field are committed to a sustainable development approach to meet today’s societal, economic, and environmental challenges. On the other hand, consumers are increasingly aware of their health, thus increasing the demand for truly effective, sensory, and safe cosmetic products. The needs are also directed toward products made with environmentally friendly, organic, and safe ingredients, such as natural and organic cosmetics (NOCs), i.e., free of irritating agents, synthetic detergents, and lipid-free, especially those for niche markets considered the new trend for sustainable beauty [1,2]. This category includes cosmetic ingredients in demand, such as bio-oils or bio-extracts, mainly for skin cleansing. Referring to the day/night skin-cleansing cosmetics, four can be mentioned: (1) liquid soaps, (2) synthetic detergent bars/liquid cleansers, from the category of syndet, (3) antimicrobial gels/soaps, and (4) lipid-free lotion cleansers, such as micellar ones [1]. Wang et al. revealed that cosmetic ingredients usually penetrate/permeate the skin and reach the blood and lymph vessels through the lipidic matrix (stratum corneum, SC), sweat gland pores, or hair follicles with their associated sebaceous glands [3]. Several factors play an important role in these processes, such as compound lipophilicity, the thickness and composition of the SC, the exposure period, the amount of topically applied product, the concentration of target compounds, occlusion, and so on [4]. It is important to point out that certain chemical-based cosmetics used for a long period may contain ingredients that are considered harmful to human health. For instance, there are several examples of dangerous ingredients that have been restricted or forbidden in cosmetics, including carcinogens such as N-nitroso-diethanolamine, formaldehyde, preservatives, hormone-disrupting chemicals, antibiotics, phthalates, phenolics, heavy metals, and even some surfactants and fragrances [5]. In addition, the term ‘toxic beauty’ has gained popularity in describing the potentially harmful impact that some chemicals, including microplastics (MPs) present in cosmetics and personal care products (whether applied topically or unintentionally eaten), may have on people’s health [6].

MPs have come under the spotlight because these tiny, insoluble, and resistant-to-degradation particles, often found as polymer mixtures with additives or impurities, are constantly present in the environment, generating environmental pollution and human health risk [7,8,9,10,11]. The definition suggested by the European Chemical Agency (ECHA) for microplastic highlights the polymer particles ‘where ≥1% *w*/*w* of particles have (i) all dimensions 0.1 μm ≤ x ≤ 5 mm, or (ii) a length of 0.3 μm ≤ x ≤ 15 mm and length to diameter ratio of >3′ [12]. The sources through which these microplastics are present in various cosmetic formulations can be accidental and/or intentional. The exfoliating beads/particles are added in beauty and care products (i.e., toothpaste, cleansing facial or body scrubs) [13,14,15]. As a general convention, in cosmetics, the term ‘microplastic’ refers to all types of plastic particles intentionally added to personal care and cosmetic products PCCPs (e.g., microbead, glitter, etc.) and continues to evolve according to ongoing scientific research [16,17]. MPs can affect human health mainly if they are ingested or inhaled. Therefore, recent studies [8,10,18] reported that microplastics act as ‘carriers’ for different emerging contaminants (ECs), such as heavy metals (HMs), polycyclic aromatic hydrocarbons (PAHs), polychlorinated biphenyls (PCBs), dioxins and furans, and so on due to the ingredients’ potential to attract and absorb this kind of contaminant, like magnets.

According to Leslie et al. [19], the presence of microplastics was quantified in human blood. These MPs can spread to other areas of the human body once they enter the bloodstream. The human immune system is not prepared to handle non-biodegradable items but can have a protective response. Inflammation may result from the immune system’s ongoing attacks on these MP particles, which can be carriers for other harmful substances. Chronic inflammation is linked to several illnesses, including cancer and autoimmune diseases [20,21,22]. The concerning issue is the presence of MPs in the human placentas already revealed by Ragusa et al. [23] and then sustained by Amereh et al. [24] and Garcia et al. [25] in their recent studies.

It is well-known that the skin is exposed to environmental stressors and impurities such as makeup, pollution, or sunscreen throughout the day. This process directly impacts the skin: it causes oxidative stress that damages the elastic and collagen fibers in the dermis and affects the mechanical properties of the skin (such as elasticity) [26,27]. Thus, the natural barrier is weakened, hydration is less effective, and inflammation intensifies. Thus, the so-called vicious circle of normal/sensitive skin appears. On the other hand, hygiene is considered the first step in any skincare routine, and its goal is to remove impurities. Rinse-off products have become increasingly popular because they provide thorough cleansing and a pleasant sensation during use [26].

Cosmetics designed to cleanse the skin (liquid soap or micellar cleansing products) commonly contain surfactants that lower the skin’s surface tension and thus facilitate the removal of dirt. Soap-based and synthetic (i.e., syndet) products are the two main categories of skin cleansers. Apart from these products that disrupt the skin barrier related to dissolving lipids and changing pH, micellar-based cleansers (i.e., micellar waters or oils) can be used for skin cleansing. Based on the data reported above, this research aims to investigate and quantify the possible presence of microplastics in usual skin-cleansing products, the most popular brands from the market, given the increased interest of humans in using microplastics-free products, which could represent an alternative to reduce the cause or risk of the aging process [28] or trigger/amplify certain diseases. The quantification of these microplastics was investigated by optical microscopy correlated with the micro-Fourier transform infrared spectroscopy (micro-FTIR) to determine the number, color, shape, size, and chemical composition of MPs. The obtained data related to the morphology and chemical composition of MPs were correlated with the chronic daily exposure to assess the potential health risk induced by the usage of the analyzed samples. Statistical analysis was used for the prediction of the occurrence of MPs in skin-cleansing samples in terms of morphology, chemical composition, and other factors (i.e., brand, packaging, etc.).

## 2. Materials and Methods

### 2.1. Materials and Reagents

The reagents used in the analyses were of HPLC grade (Sigma-Aldrich, Saint Louis, MO, USA; Merck, KGaA, Darmstadt, Germany), including sodium dodecyl sulfate (≤98.5%) and hydrogen peroxide 30%. All the materials used for additional research were thoroughly cleaned and sanitized (i.e., ultrapure water and nitric acid 2%), and they were also heated to 100°C for 48 h in a Venticell^®^ forced convection oven (BMT Medical Technology, Brno, Czech Republic). To prevent unintentional contamination (including ultrapure water) or potential interference in chemical analyses (i.e., with foreign microparticles), all materials were cleaned with ethanol 70% according to the procedure described by Zhang et al. [29]. Filter paper with a 12–15 µm porosity, VWR^®^ Grade 413 (VWR International, Radnor, PA, USA), was used to filter the skin-cleansing samples. Additionally, every filter and reagent was examined one by one. Optical microscopy confirmed that three random filters were free of MP traces and other materials or fibers. Prior to proceeding with skin-cleansing sample filtration, all possible sources of contamination were eliminated.

Cosmetics in the cleansing/skincare category were randomly chosen from specialized stores, taking into account customer preference, brand, cost, and best-sellers, according to a previously prepared questionnaire. These cleansers include 10 samples of liquid soap, 6 samples of micellar water, and 6 samples of micellar cleansing oil, according to the data presented in Table S1.

### 2.2. Sample Preparation

Chemical digestion is the most well-known method applied in isolating microparticles from various matrices, facilitating the filtration of samples, and decreasing the chances of filter clogging. The microparticle isolation protocol was established according to research conducted by Banica et al. [27] and Radulescu et al. [30], with three key steps: (i) pretreatment of samples using HPLC grade reagents; (ii) proper digestion; (iii) filtration. First, to 10 mL of liquid soap samples, 500 mL of ultrapure water was added. The mixture was stirred for 5 min, and after that, it was filtered. In the case of micellar water samples, the MPs isolation was achieved by using a volumetric mixture of 20 mL of sample and 20 mL of hydrogen peroxide, respectively, in a 1:1 ratio. After five minutes of stirring and a resting period of 30 min, the obtained samples were filtered. Regarding micellar cleansing oils, the microplastic isolation protocol is more elaborate. To analyze the micellar cleansing oil samples, a mixture of 2 mL of the sample and 1 g of sodium dodecyl sulfate was prepared, and then 500 mL of ultrapure water was added. The obtained mixture was stirred for 5 min at a speed of 150 rpm to obtain a homogeneous mixture. After a resting period of 30 min, the mixture was subjected to an ultrasonic treatment at a temperature of 40 °C for 30 min to facilitate the dispersion of the components. The samples were filtered after heating the mixtures to a temperature of 60 °C.

### 2.3. Analytical Investigation

#### 2.3.1. Optical Microscopy

Optical microscopy (OM) was used to identify the isolated microparticles from three skin-cleansing product categories. In this regard, the samples were examined in transmitted and reflected light using a Primo Star and Stemi 2000-c optical microscope (Carl Zeiss, Jena, Germany). The Axiocam 105 digital video camera (Carl Zeiss, Jena, Germany) and Zen software ZEN 2012, blue edition, Version 1.1.2.0 were used to acquire the final images. In addition, optical microscopy helped in microparticle identification, quantification, isolation, and characterization in terms of size, form, color, and number.

#### 2.3.2. Micro-Fourier Transform Infrared Spectroscopy

Fourier transform infrared spectroscopy (FTIR) is a non-destructive, vibrational analytical technique used to identify functional groups in organic, polymeric, and inorganic samples [7,8,9]. This technique uses infrared light to scan samples to determine the chemical structure. The absorbed radiation is converted into rotational or vibrational energy by the sample molecules. This spectral signature is obtained after processing the raw data by Fourier transformation and is presented as a spectrum in a certain interval, representing the molecular fingerprint of the sample. The wavelength of each IR vibrational absorbance peak is representative of the intrinsic chemical bonds of the analyzed molecule. Every molecule or chemical structure produces a unique fingerprint of the corresponding functional groups, making FTIR analysis an excellent tool for chemical characterization. Micro-FTIR analysis provides a non-destructive characterization of samples that are too small to be chemically analyzed by conventional FTIR [7,8,9,27]. Apart from qualitative investigation, FTIR and micro-FTIR techniques can quantify the concentration of functional groups from the sample by comparing the intensities of the vibrational bands measured on a sample with calibrated curves. Micro-FTIR characterization can be performed in transmission or reflection light mode. Equipped with a diamond ATR (Attenuated Total Reflection) crystal accessory and a Hyperion 3000 microscope, the Vertex 80v FTIR spectrometer (Bruker, Berlin, Germany) performs analysis of microplastics in three modes: transmission, reflection (ATR), and micro-FTIR in imaging mode.

Hyperion microscope is characterized by a 600–7500 cm^−1^ spectral range, 0.2 cm^−1^ spectral resolution, and ±1 µm accuracy. The design allows the highest performance for visual examination and infrared analysis of any sample to be obtained. An air background spectrum was recorded using 12 co-added scans, with sample spectra subsequently recorded with 32 co-added scans. Signals were collected with a high spatial resolution of a few micrometers. FTIR imaging spectra were baseline corrected, processed, and analyzed using Bruker OPUS v.7.5 software spectra library (Bruker, Berlin, Germany), under 19% relative humidity.

### 2.4. Statistical Analysis

The recorded data were analyzed using IBM SPSS Statistics software v.26 (SPSS Inc., Chicago, IL, USA, 2011). To understand data complexity, Pearson’s correlation analysis and Hierarchical Cluster Analysis (HCA) were performed. Statistical analysis is the most used tool to explore the similarities between samples, contributing to data organization by eliminating redundancies and detecting anomalies. The *p*-value < 0.05 was considered statistically significant.

### 2.5. Assessing Exposure Method to Microplastics

Loprieno [1] mentioned in his study that before evaluating the health risk of the finished product, the degree of risk and the route of exposure of the consumer must be established. Thus, he stated that several factors must be taken into account, including the following: (i) the class of the cosmetic product; (ii) the method of applying the product (massage, application and rinsing, spraying, etc.); (iii) the concentration of the ingredient in the product; (iv) the amount applied for each use; (v) frequency of application; (vi) the total surface of the skin that comes into contact with the product; (vii) the place of contact (mucous membranes, sunburned skin); (viii) duration of contact (if the product is rinsed or not); (ix) wrong use of the product; (x) the nature of the consumer (adults, children, people with sensitive skin, etc.); (xi) the amount likely to enter the body or reference doses (results from bioavailability studies); (xii) the proportion of consumers; (xiii) applications on skin exposed to sunlight.

In the absence of any equations/calculation formulas that allow the evaluation of the health risk induced by the use of cosmetic products containing microplastics, the authors resorted to adapting the formulas that establish the daily chronic dose of exposure to heavy metals induced by contaminated soils [31,32,33,34] taking into account a large part of the previously mentioned factors, as the following equations:

(1) $$D_{der}=\frac{C\cdot SA\cdot ED\cdot EF}{BW\cdot AT}\cdot CF$$

(2) $$D_{ing}=\frac{C\cdot IR\cdot ED\cdot EF}{BW\cdot AT}\cdot CF$$

(3) $$D_{inh}=\frac{C\cdot InhR\cdot ED\cdot EF}{BW\cdot AT\cdot PEF}$$

where

D_der_ = chronic daily exposure dose through dermal absorption, expressed in n·kg^−1^·day^−1^;

D_ing_ = chronic daily exposure dose by ingestion, expressed in n·kg^−1^·day^−1^;

D_inh_ = chronic daily dose of inhalation exposure, expressed in n·kg^−1^·day^−1^;

C = concentration of microplastics, expressed in n·kg^−1^ or n·L^−1^;

ED = duration of exposure, expressed in years (for adults ED = 20 years);

EF = exposure frequency, expressed in day·year^−1^(for adults EF = 365 day·year^−1^);

BW = body mass, expressed in kg (for adults BW = 70 kg);

AT = the average exposure time, expressed in days (for adults AT = 7300 days);

SA = exposed surface area, expressed in cm^2^ (for adults SA_total_ = 17,500 cm^2^ and

SA_face-neck_ = 787.5 cm^2^);

CF = conversion factor (CF = 1 × 10^−6^);

IR = ingestion rate, expressed in mg·day^−1^ (for adults IR = 100 mg·day^−1^);

InhR = inhalation rate, expressed in m^3^·day^−1^ (for adults InhR = 20 m^3^·day^−1^);

PEF = emission factor of the particles, expressed in m^3^·kg^−1^ (PEF = 1.36 × 10^9^ m^3^·kg^−1^).

The total daily chronic exposure dose is obtained by summing the three determined doses according to Equation (4):

(4) $$D_{total}=D_{ing}+D_{inh}+D_{dermal}$$

In the absence of standard values for daily reference doses (reference dose—RfD) or for cancer slope factors (Cancer slope factor—CSF) established by competent bodies/authorities (for example: World Health Organization—WHO, U.S. Food and Drug Administration—FDA, U.S. Environmental Protection Agency—US EPA, Institute of Environmental Science and Research Limited—ESR, etc.), the risk to human health cannot be calculated as it is obtained either by dividing the chronic dose by the RfD or by multiplication with CSF (in the case of polymers with carcinogenic potential).

## 3. Results and Discussion

### 3.1. Optical Microscopy

Optical microscopy revealed the retained microparticles on the filter’s surface after isolation (Figures S1–S3). Table 1, Table 2 and Table 3 show the quantification and classification of microparticles according to color (i.e., black, blue, grey, red, green, purple, turquoise, and brown). Values are expressed as microparticles per liter (i.e., n·L^−1^).

According to optical microscopy, the microparticles identified in all ten liquid soap samples were quantified; there was a total number of 61,000 and they had different colors (black, blue, grey, red, green, purple, and turquoise). The highest number of microparticles was present in samples LS5 and LS7. In the aspect of color, it states that black predominated (24,000 microparticles) and was identified in all liquid soap samples, blue (27,000 microparticles) was identified in nine of them, grey (4000 microparticles) in three samples (LS2, LS6, and LS7), red (3000 microparticles) in samples LS3 and LS7, and green, purple, and turquoise (1000 microparticles) were detected in sample LS1, LS5, and LS6, respectively (Table 1).

On the other hand, various microparticles were recorded in other cleanser samples, such as micellar water samples (Figure S2).

The same overall number (61,000 microparticles) was obtained as in the case of liquid soap samples. The predominant black and blue colors were identified in all six samples in an abundance of 37,000 and 14,000 microparticles, respectively. In sample MW1, 4000 red microparticles were detected. About 2000 fines grey microparticles were identified in samples MW3 and MW4. Purple and turquoise-colored microparticles were identified in MW4 and MW1, respectively (Table 2).

In the case of six micellar cleansing oil samples (Table 3), a total of 35,000 colored microparticles were quantified. The color-spreading was as follows: black (20,000 microparticles) in MCO1–MCO6 samples, blue (11,000 microparticles) in five micellar cleansing oil samples, red (2000 microparticles) in two samples (i.e., MCO1 and MCO4), and grey and purple (1000 microparticles) in sample MCO4.

### 3.2. Micro-Fourier Transform Infrared Spectroscopy

**Figure 1:**
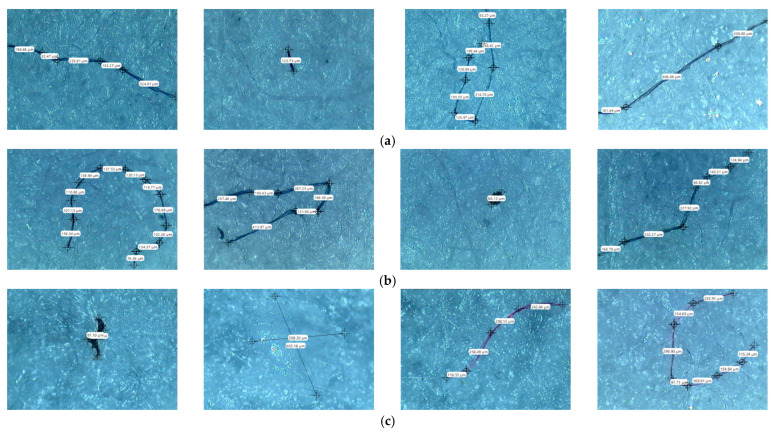
Measurement sequence of MPs identified in the analyzed samples (few examples fiber/fragments): (**a**) Liquid soap; (**b**) Micellar water; (**c**) Micellar cleansing oil.

**Figure 2:**
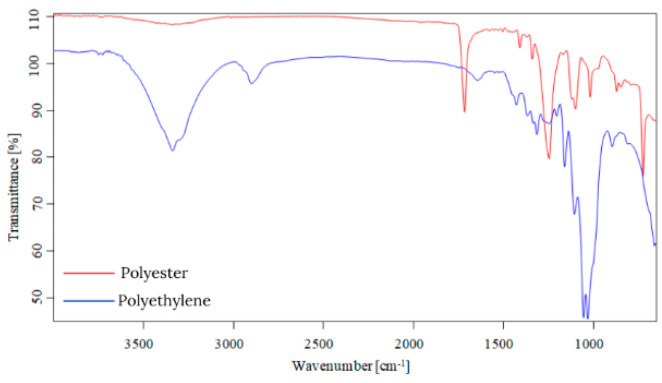
Overlapping spectra of singular polymers identified in liquid soap samples.

**Figure 3:**
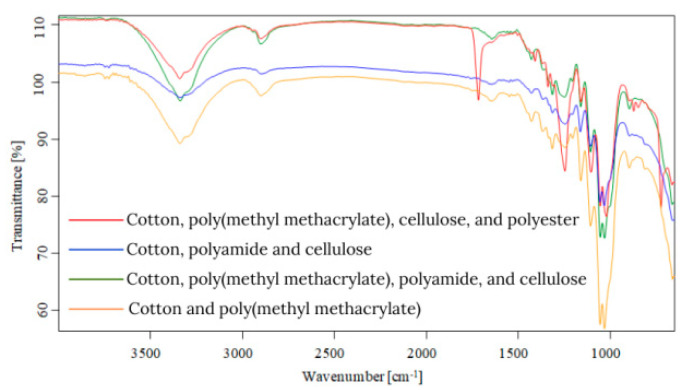
Overlapping spectra of synthetic mixtures identified in liquid soap samples.

**Figure 4:**
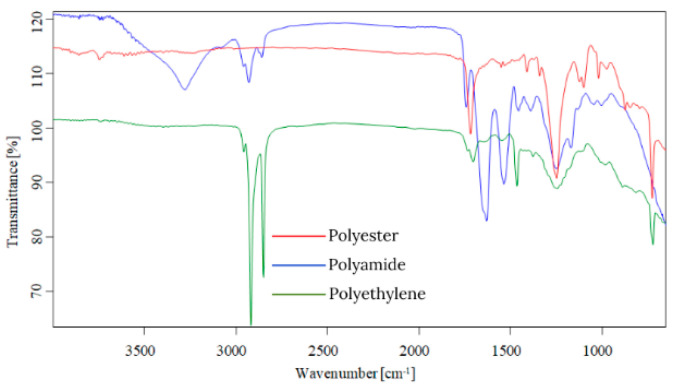
Overlapping spectra of singular polymers identified in micellar water samples.

**Figure 5:**
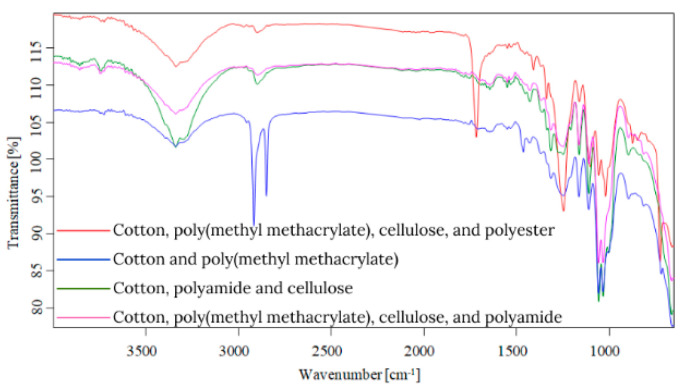
Overlapping spectra of synthetic mixtures identified in micellar water samples.

**Figure 6:**
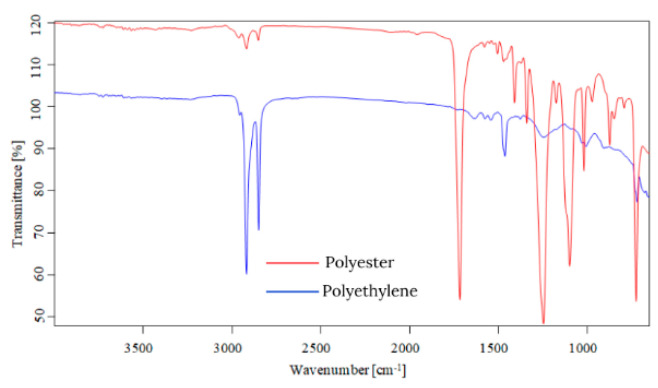
Overlapping spectra of polymers identified in micellar cleansing oil samples.

**Figure 7:**
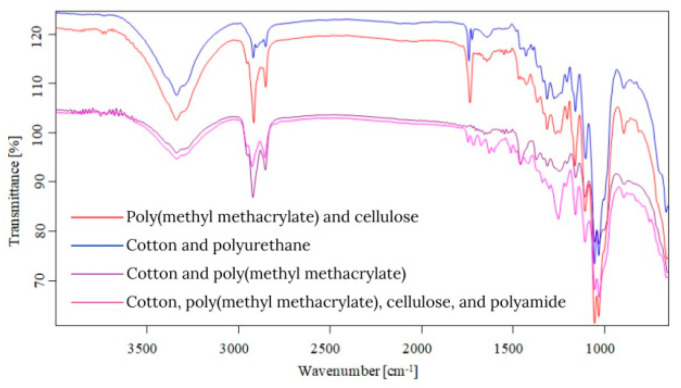
Overlapping spectra of synthetic mixtures identified in micellar cleansing oil samples.

Starting from the first data obtained by optical microscopy, micro-Fourier transform infrared spectroscopy is the next step in the investigation of microparticles. Even if transmittance-micro-FTIR mapping (micro-FTIR) of microplastic samples from skin-cleansing cosmetic products is still limited in terms of fine-scale chemical characterization, the preliminary research led to surprising results related to the presence and structure of MPs. In this regard, preliminary investigations related to MPs’ composition, in a minimum of three points, revealed several fibers and fragments with different sizes and shapes and various chemical compositions (Tables S2–S4). In addition, by using the OPUS v.7.5 software spectra library provided by Bruker, Germany, with more than 50,000 spectra in the library, it was possible to identify the proper composition of microparticles (synthetic or mixture, natural). This primary information led to eliminating the microparticles that had a natural chemical composition (i.e., wool, cotton, hemp, flax, viscose, kenaf, jute). Tables S2–S4 present the micro-FTIR images (with a mapping in minim three points), the chemical composition provided by OPUS v.7.5 software spectra library (Bruker, Berlin, Germany), and the shapes of fibers and fragments of MPs. In the case of fibers identified as a mixture (i.e., natural and synthetic), in Tables S2–S4, the % chemical composition at a minimum of three points was provided. In the case of fragments, besides chemical composition, the shape of fragments was revealed (i.e., irregular, angular, and elongated). Micro-FTIR mapping provided high-quality polymeric structure identification, even if it was time-consuming. Apart from the aforementioned, micro-FTIR was used for MPs size determination (Figure 1). Using micro-FTIR imaging, a complete characterization (chemical and morphological, Tables S2–S4, and Figure 1) of MPs was achieved, and the obtained results are reliable and reproducible. Differences between the micro-FTIR spectra of MPs isolated from cleanser cosmetic samples can be observed relative to a series of spectral regions/peaks highlighted in Figure 2, Figure 3, Figure 4, Figure 5, Figure 6 and Figure 7. After correction for the background spectrum was achieved, all analyzed spectra showed different weak, medium, and strong vibrational frequencies according to the FTIR imaging. In spectroscopic terms, the weak, medium, and strong peaks (intensity) and wavenumbers allow the assignment of functional groups from organic/polymeric compounds [35].

The measurement sequence of the identified microparticles (Figure 1) was made using the OPUS v.7.5 software, for 108 microparticles from the liquid soap samples, 99 microparticles from the cleansing oil samples, and 86 microparticles from the micellar water samples.

Table 4 shows the classification of MPs according to their size and fiber or fragment type. Five categories were established: below 50 µm; in the range of 50 and 100 µm; between 100 and 500 µm; between 500 and 1000 µm, and over 1000 µm.

Depending on the size of the identified MPs, the samples were grouped into five classes: <50 µm, which includes nine MPs, in the range of 50 and 100 µm (13 MPs), ranging between 100 and 500 µm (143 MPs), between 500 and 1000 µm (99 MPs), and >1000 µm (29 MPs). Table 4 shows MPs’ classification according to shape (i.e., 78 fibers and 30 fragments identified in liquid soap, 44 fibers and 44 fragments in micellar water, and 61 fibers and 39 fragments in micellar cleansing oil).

In addition, MPs’ composition recorded by micro-FTIR is centralized in Table 5 in terms of natural or synthetic polymers.

According to Table 5, from the category of natural compounds identified from the 296 MPs, cotton was found in 174, cellulose in 87, flax in 28, and wool in five. Five synthetic polymers (i.e., poly(methyl methacrylate), polyamide, polyester, polyethylene, polyurethane) were detected in the composition of MPs from analyzed cleansers, as follows: poly(methyl methacrylate) in 46 MPs, polyamide in 14 MPs, while polyester, polyethylene, and polyurethane were found in five, four, and two MPs, respectively. Also mentioned were 26 polymer structures included in the ‘other polymers’ category (Table 5).

FTIR spectra analysis of polymers was recorded in the range of 600–4000 cm^−1^ as a complementary tool for polymeric structures. Hidden information contained in the complex and overlapping peaks can be used for a good interpretation of the chemical structure of MPs. In this regard, Bredacs et al. [36] revealed spectral data of polyethylene in terms of chain distribution and length. In this regard, the recorded signals ranging from 2930 to 2915 cm^−1^ show the sum of the C-H stretching assigned to polyethylene structures, according to data shown in Figure 2, Figure 3, Figure 4, Figure 5, Figure 6 and Figure 7. On the other hand, the stretching vibrations of the CH_2_ group in polyethylene presented as strong peaks were in the range of 2845 and 2917 cm^−1^ (more visible in Figure 4 and Figure 6, for selected MW2.1 and MCO3.1 samples, respectively,). In addition, the weak intensity peaks around 719–730 cm^−1^ were assigned to the CH_2_ stretching vibration in polyethylene, at which a CH_2_ bending vibration mode as a medium intensity peak at 1473 cm^−1^ (Figure 4 and Figure 6) was added. According to Figure 2, Figure 3, Figure 4, Figure 5, Figure 6 and Figure 7, the peaks around 1100 cm^−1^ were attributed to the O-C-C stretch of the ester functional group from polyesters, while the peaks that fell from 1310 to 1250 corresponded to aromatic groups of polyesters (i.e., C-C-O linkage in red spectra from Figure 2, Figure 3, Figure 4, Figure 5, Figure 6 and Figure 7). Generally, the C=O group and two C-O stretches from polyesters revealed a pattern of three intense peaks in the vicinity of 1700 cm^−1^ for the carbonyl stretches; for aromatic carbonyl groups, the tendency was to be 30 cm^−1^ lower than for saturated carbonyl groups due to the conjugation, and the strong peaks around 1200 cm^−1^ and ~1100 cm^−1^ were attributed to the two C-O stretches (Figure 2, Figure 3, Figure 4, Figure 5, Figure 6 and Figure 7). The small singular or in-mixture polyester fibers were observed in liquid soap, water micellar, and micellar cleansing oil samples in terms of strong peaks around 687–698 cm^−1^ assigned to C-H symmetric stretching from the aromatic ring of polyester [27,37].

The N-H bending and C-N stretching of amidic groups from polyamides (nylon) can be assigned to the peaks around the values 1265–1278 cm^−1^ and 1538–1342 cm^−1^. In addition, the peaks around 2858 and 2932 cm^−1^ were attributed to the CH stretching of polyamides. The signals around 1634 cm^−1^ were assigned to C=O stretching from polyamides, while the weak peaks from 1190, 1373, and 1466 cm^−1^ were assigned to CH_2_ bending vibration as well [7,8,9,27,38,39]. The weak/medium peaks around 1720 cm^−1^ and 750 cm^−1^ were assigned to the stretching and bending vibrations of the C=O group, respectively (Figure 3, Figure 5 and Figure 7), derived from poly(methyl methacrylate) polymers [7,8,9,27,39]. In addition, the medium peaks from 964, 2950, and 2990 cm^−1^ were attributed to the bending and stretching vibration of the C-H group from poly(methyl methacrylate) [40,41]. The C-O stretching from poly(methyl methacrylate) was also identified in the synthetic mixture (Figure 3, Figure 5 and Figure 7) around 1140 cm^−1^ and 1235 cm^−1^ as weak/medium peaks, being confirmed by Jung et al. [40] in their research. In the case of the polyurethane polymer from the synthetic mixture (Figure 7), the representative C(=O)O stretching group was assigned to the medium peak of 1221 cm^−1^.

Weak- and medium-intensity bands have been highlighted in all recorded spectra. The region of 1032–891 cm^−1^ is that of the C-H deformation in the plane, and the strong intensity bands recorded can be due to C-O stretching. From the analytical point of view, the C-H stretching region (i.e., from 2800 to 2950 cm^−1^) is more meaningful as several distinct and assignable absorption differences are visible in the spectra (strong and medium peaks, Figure 2, Figure 3, Figure 4, Figure 5, Figure 6 and Figure 7). On the other hand, several changes in vibrational frequency resulted, such as spectral shifts due to different inter/intramolecular interactions that may influence the structure, e.g., the steric effects. Thus, it can be seen from Figure 2, Figure 3, Figure 4, Figure 5, Figure 6 and Figure 7 that several values of the obtained FTIR spectra, expressed as wavenumbers, related to the symmetric CH_2_ stretching mode are instead shifted to higher wavenumbers [7,8,9]. This is assigned to overlap with the symmetric CH_3_ stretching modes; the overlapping of CH_3_ and CH_2_ therefore can appear as broadband [7,8,9,27] according to spectra presented in Figure 2, Figure 3, Figure 4, Figure 5, Figure 6 and Figure 7. The most prevalent polymers found in cosmetic skin-cleansing products were poly(methyl methacrylate) > polyamide > polyethylene > polyester > polyurethane (i.e., singular polymers or in mixtures, Tables S2–S4). Other identified chemical structures were assigned to additives, plasticizers, natural and non-plastic synthetic fibers, atmospheric deposition, and other inputs.

### 3.3. Statistical Analysis

The methodology used to analyze the presence and concentration of microplastics in cosmetic products involves a systematic approach based on data collection, processing, and applying appropriate statistical techniques. This analysis mainly aims to identify distribution patterns, variations between product types, and the correlation between factors contributing to microplastics and fiber type presence. Samples were taken from three categories of cosmetic products (liquid soap, micellar water, and micellar cleansing oil) for each product, recording information on the product brand, packaging type, storage conditions, number of microplastic particles identified, and their shape and size.

The Pearson correlation coefficient establishes the relationship between the ‘fiber type’ and the ‘occurence source’ (Table 6) of the microparticles analyzed. A moderate relationship was highlighted for micellar water (R = 0.659), and a relationship of dependence was strong in the case of liquid soap (R = 0.808) and micellar cleansing oil (R = 0.770).

The linear regression method explains the connection between ‘fiber type’ and ‘occurence source’. In order to establish the potential sources of occurrence, the authors had to understand the technological process of obtaining the products (micellar water, micellar cleansing, and liquid soap). Together with the specialized staff (operators and preparers from the industry), the potential sources of occurrence were allocated (the work equipment—gowns, caps, protective masks, etc.,—the packaging in which the preparations are made, the product sales arrangement, and the purity of the substances).

The coefficient of determination R^2^, in the case of micellar water, was 0.434, which represents the variation in the dependent variable in a proportion of 43.4% compared to the independent variable (Table 7). Simultaneously, this coefficient was also determined in the case of the other two products, where R^2^ = 0.653 and 0.593.

**Figure 8:**
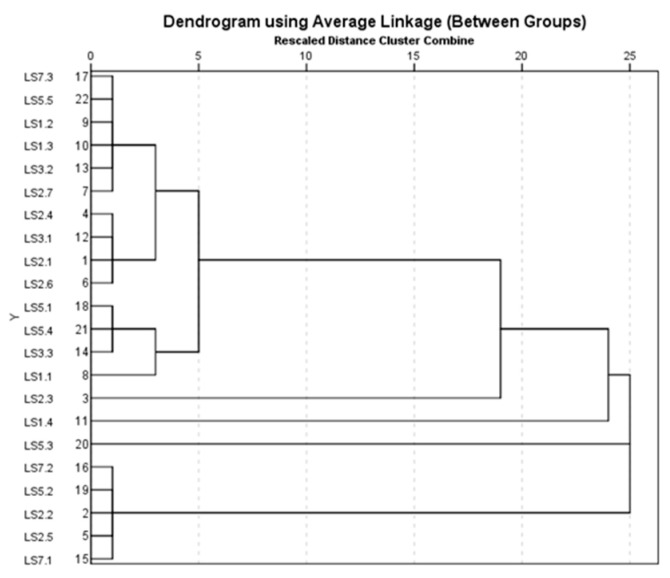
Grouping of liquid soap samples according to composition.

**Figure 9:**
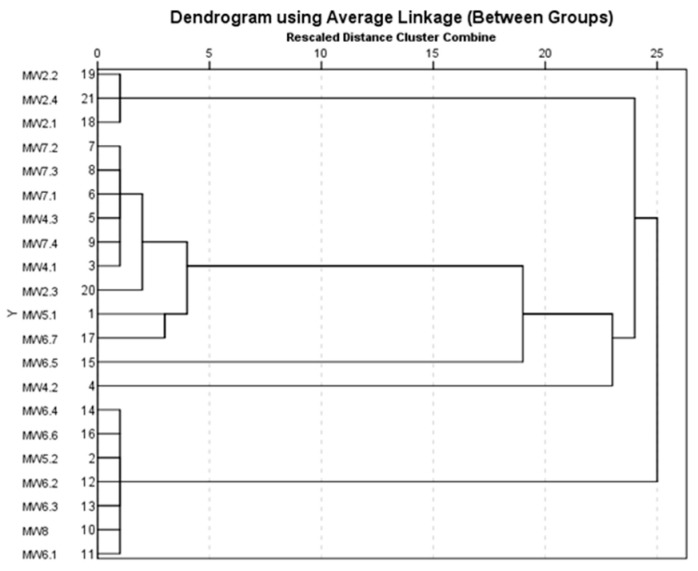
Grouping of micellar water samples according to composition.

**Figure 10:**
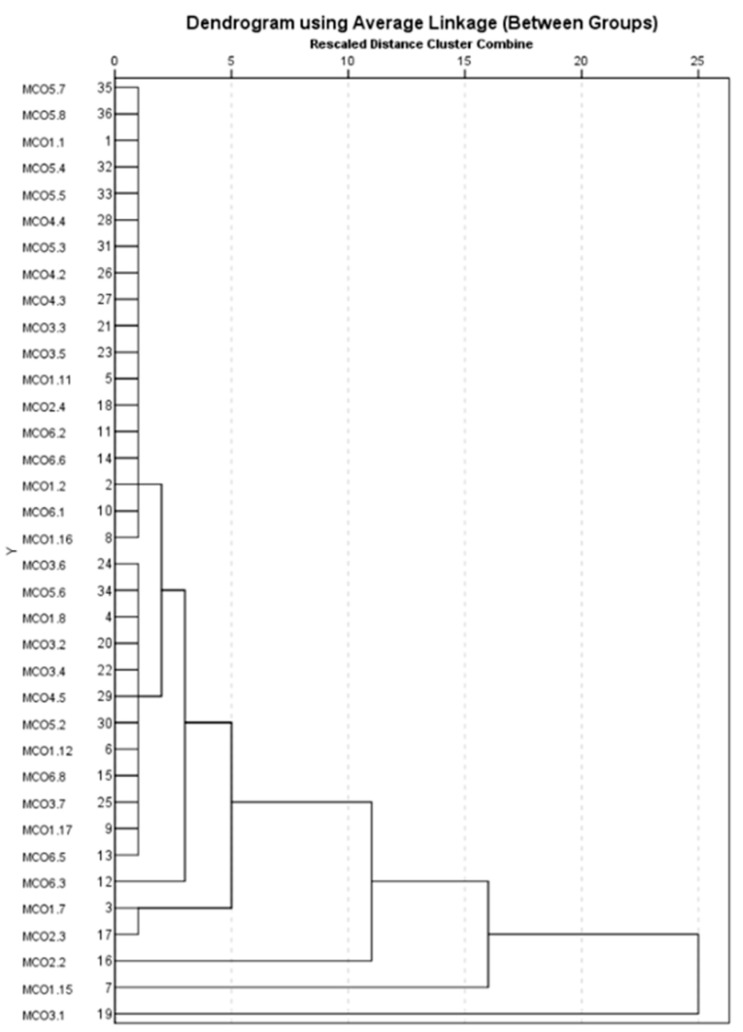
Grouping of micellar cleansing oil samples according to composition.

The cluster analysis performs a classification of the samples evaluated in terms of the composition of identified microplastics (Figure 8, Figure 9 and Figure 10). In this regard, Figure 8 shows the grouping of liquid soap samples into eight classes, of which four are represented by synthetic mixtures: cellulose, cotton, poly(methyl methacrylate), and polyamide; cotton with poly(methyl methacrylate); cotton and poly(methyl methacrylate); cotton with polyester; cellulose, cotton, and polyamide; cellulose, cotton, and polyamide; cellulose, cotton, poly(methyl methacrylate), and polyester. Four classes were also identified in which the polymers were 100% (polyester, polyamide, polyethylene, and poly(methyl methacrylate)). In the case of liquid soap samples, four classes of mixtures and three classes in which polymers were identified at 100% can be observed (Figure 9). The synthetic mixture class was composed of the following: cotton with poly(methyl methacrylate); cellulose, cotton, poly(methyl methacrylate), and polyamide; cotton, nylon, and poly(methyl methacrylate); cotton with polyester; cellulose, cotton, and polyamide; cellulose, cotton, poly(methyl methacrylate), and polyester. The polymers identified at 100% were polyethylene, poly(methyl methacrylate), and polyester.

The cluster grouping of the micellar cleansing oil samples (Figure 10) shows five clusters in which the composition is a synthetic mixture: cellulose, cotton, poly(methyl methacrylate), and polyamide; cotton with polyurethane; cotton with poly(methyl methacrylate); cotton, nylon, and poly(methyl methacrylate); cotton with polyester; and two clusters in which the composition of the analyzed samples was 100% polymer, respectively, polyester, polyethylene.

### 3.4. Assessing Exposure Paths to Microplastics

The cosmetic product market is in continuous development, both in terms of product categories and brands that produce these products. In the last 50 years, various regulations, legislations, and safety assessment guidelines for cosmetic products have been formulated; among these, the Council Directive of 27 July 1976, on the approximation of the legislation of the member states regarding cosmetic products issued by the Council of the European Communities 76/768/EEC [42] and Notes of guidance for testing of cosmetic ingredients for their safety evaluation (SCCS/803-5190) issued by the Society of Cosmetic Chemists in 1990—revised by Scientific Committee on Consumer Safety in 2023 as SCCS/1647/22 can be mentioned [43]. Directive 76/768/EEC specifies that “Cosmetic products means any substance or preparation intended to be applied to various external parts of the human body (epidermis, hair, nails, lips, and external genital organs) or the teeth and oral mucosa, in the exclusive or main purpose of cleansing, perfuming or protecting them to keep them in good condition, altering their appearance or correcting body odors.” [42].

At the European level, the European Chemicals Agency (ECHA) has estimated that 3800 tons of microplastics end up in the environment due to the daily use of cosmetic products [44]. To reduce this category of pollutants, the European Commission developed Commission Regulation (EU) 2023/2055 of 25 September 2023, amending Annex XVII to Regulation (EC) no. 1907/2006 of the European Parliament and the Council on the Registration, Evaluation, Authorization, and Restriction of Chemicals (REACH) as regards microparticles of synthetic polymers [45]. Taking into account the fact that the research presented in this manuscript was started as early as 2021—that is, before the publication of Regulation 2023/2055—it is mentioned that even up to this date, there were no normative acts that allow the evaluation/calculation of the risk to human health because of the use of cosmetic products. Reducing or banning the use of microplastics in cosmetics is very important, but it is a long-term process. Regulation EU 2023/2055 specifies that it is mandatory to reduce the proportion of added microplastics from 1% to 0.01%, as follows: (1) until 2029 for microparticles of synthetic polymers intended for use in perfume encapsulation; (2) until 2027 for rinse-off products; (3) until 2035 for lip products; (4) until 2029 for products without rinsing; (5) until 2028 for detergents, etc. [45].

According to research published by Loprieno [1], the assessment of the safety of any cosmetic product is performed by referring to the method of use and the compounds contained in it, but taking into account all types of exposure, because in this way, it determines the amount of substance that can be ingested, inhaled, or absorbed through the skin or mucous membranes [1]. Micro- and nanoplastics are considered hidden ingredients of cosmetic products, as they are intentionally added in the manufacturing process but do not appear in the list of ingredients [44]. The purpose of adding microplastics to cosmetic products is to obtain an exfoliating effect, to stabilize the product (prevents the separation of emulsions or even induces the emulsification process), to modify the texture of the finished product, to obtain an opacifying, mattifying, or shimmering product, or to form a film on the skin, hair, or nails [46,47,48]. Starting from European legislation and scientific research, it is important to highlight again the primary route of exposition and uptake of MPs: ingestion (via the digestive system), inhalation (via the lungs), and absorption, i.e., direct skin contact. The exposure involves the MPs’ inhalation and direct contact with the skin. It is well known that the primary target of inhaled MPs is the respiratory epithelium [49].

According to a study recorded by Amato-Lourenço et al. [50], microplastic fibers or fragments were evidenced in human lung tissue during autopsies, and this can be a genuine concern about the risks developed in different illnesses, with a connection even to tumor diseases. On the other hand, Persoons et al. [51] revealed that the workers exposed to a large amount of airborne polystyrene particles were monitored; the presence of polymers in the urine of human subjects was linked with the inhalation/absorption process of MPs. Differently, the absorption/penetration of MPs into the skin is analyzed. The skin is the protective barrier of the human body and could be potentially permeable to different microparticles present mainly in cosmetics [52]. Several types of research revealed that MPs can penetrate the stratum corneum through exfoliated skin [53], skin injuries [17], or through upper sections of hair follicles [54]. Taking into account these hypotheses, sustained by biological and physicochemical investigations, it can be suggested that some cosmetic cleansers from the category of skin care, such as liquid soaps, micellar waters, and micellar cleansing oils that accidentally contain polymeric particles (i.e., micro or nanoplastics), can represent a health risk in terms of inhalation or absorption. The ingestion of cosmetic skin care cleansers by humans may be possible accidentally and mainly by the gastrointestinal tract during shower washing processes [53].

Worryingly, microplastics in humans may accelerate the aging process by increasing oxidative stress in the body. Oxidative stress occurs when free radicals accumulate, causing damage to cells and tissues. This damage can particularly affect mitochondrial function, which is responsible for energy production in cells [55]. When mitochondrial function is impaired, it can lead to age-related diseases and reduced physical function.

In recent research, Moon et al. [55] reported that microplastics may contribute to faster aging by increasing oxidative stress and impairing the body’s ability to regenerate cells. So, on the one hand, there is a continuous concern among people regarding skin aging, and a continuous struggle to find natural cosmetic formulations, rich in antioxidants and polyphenols, and on the other hand, there is a concern that these plastic particles can accelerate the aging process of skin cells by increasing oxidative stress.

To our knowledge, until now, there have only been presumptive studies related to concerns about the risk posed by the accidental or non-accidental presence of microplastics in skin care products. There are only tentative preliminary studies on the carcinogenic effect of microplastic exposure. There is still no calculation formula established for the dose regarding the ingestion, inhalation, absorption of MPs, or even the total chronic exposure dose for all three exposure routes to determine the health risk following exposure. In this regard, in this study, for the first time, based on previous research [7,8,9,32,56], the authors calculated the doses of ingestion, inhalation, and absorption, and chronic total exposure dose to microplastics for the three skin care cleanser categories, liquid soaps, micellar waters, and micellar cleansing oils (Table 8). According to Table 8, the exposure doses through direct skin contact to MPs are higher for all micellar cleansing oil samples (>0.3 n·L^−1^·day^−1^), except for samples MCO1 and MCO4 where the absorption dose is equal to 1.0 n·L^−1^·day^−1^ and 1.25 n·L^−1^·day^−1^, respectively. In the case of the analyzed liquid soap samples, slightly higher values are observed for samples LS3, LS5, LS6, and LS7 (>0.2 n·L^−1^·day^−1^). The value obtained for the inhalation dose is very low, meaning there is a minimal risk that MPs can be inhaled from cleansing skin care products. In addition, the obtained values of ingestion are low, and the risk of ingestion is rather an accidental one during the skin-cleansing process. Therefore, absorption determines the highest exposure risk, i.e., direct skin contact.

The European recommendation is that cosmetic cleansing formulations, if they contain polyethylene microgranules, should be replaced with microcrystalline cellulose particles and mixtures, silicon dioxide particles, and castor wax, gentle and skin-friendly ecological alternatives that offer the same peeling effect. In the case of this research, it was clearly observed that the analyzed skin-cleansing products did not contain intentionally introduced microgranules with a polymeric structure but only fragments or fibers, mostly in the form of mixtures, originating non-intentionally during the production, packaging, or handling of the products. However, these findings raise concerns about the potential health risks of MPs exposure.

## 4. Conclusions

This study uses optical microscopy and micro-FTIR mapping to investigate the morphology and chemical composition of MPs from two categories of skin care products (i.e., liquid soap, micellar water, and micellar cleansing oil). This research is the first step to identifying and quantifying microplastics in personal care products, such as skin cleansers. Based on the obtained results, it can be highlighted that some polymer structures (e.g., poly(methyl methacrylate), polyamide, polyethylene, polyester, polyurethane) are present in all analyzed samples, as a singular form or in a mixture with other natural/synthetic compounds. Micro-FTIR imaging, combined with optical microscopy, was an accurate way to characterize both chemical and morphological properties and quantify microplastics in the analyzed cleanser skin cosmetics. In addition, statistical analysis provided significant correlations related to distribution patterns, variations between product types, and factors that contribute to the MPs’ presence in analyzed cleansers, taking into consideration category, brand, packaging type, storage conditions, and a few identified MPs, their shape, and their size. Next, in accordance with the information obtained through analytical and statistical methods, the exposure routes to microplastics were evaluated for the first time. The obtained results confirmed the hypotheses that micellar cleansing oil retains MPs more easily than liquid soap or micellar water. In the future, various exposure and toxicity assessments in terms of MPs are expected to assess the potential human health risk. What is known is the fact that the toxic effect of MPs depends on their ubiquitous nature and long persistence and is strongly linked with the type, size, shape, and concentration of microplastics. The research highlights the need for further research to understand the long-term consequences of MPs’ accumulation in humans and the potential health risks. Cosmetics reform is not only an obligation imposed by legislation but also an opportunity for innovation and evolution.

## Figures and Tables

**Table 1:** Optical microscopy for liquid soap samples.

Sample Code	Color and Number of Microparticles							Total [n·L^−1^]
	Black	Blue	Red	Green	Grey	Purple	Turquoise	
LS1	2000	2000	nd*	nd*	nd*	nd*	1000	5000
LS2	2000	1000	nd*	nd*	2000	nd*	nd*	5000
LS3	5000	3000	1000	nd*	nd*	nd*	nd*	9000
LS4	1000	1000	nd*	nd*	nd*	nd*	nd*	2000
LS5	3000	6000	nd*	nd*	nd*	1000	nd*	10,000
LS6	2000	4000	nd*	1000	1000	nd*	nd*	8000
LS7	4000	3000	2000	nd*	1000	nd*	nd*	10,000
LS8	1000	1000	nd*	nd*	nd*	nd*	nd*	2000
LS9	2000	nd*	nd*	nd*	nd*	nd*	nd*	2000
LS10	2000	6000	nd*	nd*	nd*	nd*	nd*	8000
Total	24,000	27,000	3000	1000	4000	1000	1000	61,000

nd*—unidentified.

**Table 2:** Optical microscopy for micellar water samples.

Sample Code	Color and Number of Microparticles							Total [n·L^−1^]
	Black	Blue	Red	Grey	Purple	Turquoise	Brown	
MW1	24,000	2000	4000	nd*	nd*	1000	2000	33,000
MW2	2000	1000	nd*	nd*	nd*	nd*	nd*	3000
MW3	2000	2000	nd*	1000	nd*	nd*	nd*	5000
MW4	3000	6000	nd*	1000	1000	nd*	nd*	11,000
MW5	3000	2000	nd*	nd*	nd*	nd*	nd*	5000
MW6	3000	1000	nd*	nd*	nd*	nd*	nd*	4000
Total	37,000	14,000	4000	2000	1000	1000	2000	61,000

nd*—unidentified.

**Table 3:** Optical microscopy for micellar cleansing oil samples.

Sample Code	Color and Number of Microparticles					Total [n·L^−1^]
	Black	Blue	Red	Grey	Purple	
MCO1	3000	6000	1000	nd*	nd*	10,000
MCO2	5000	1000	nd*	nd*	nd*	6000
MCO3	2000	1000	nd*	nd*	nd*	3000
MCO4	4000	1000	1000	1000	1000	8000
MCO5	3000	nd*	nd*	nd*	nd*	3000
MCO6	3000	2000	nd*	nd*	nd*	5000
Total	20,000	11,000	2000	1000	1000	35,000

nd*—unidentified.

**Table 4:** Sample distribution based on MPs size analyzed by micro-FTIR.

Sample	<50 [µm]	50–100 [µm]	100–500 [µm]	500–1000 [µm]	>1000 [µm]	Shape of MPs	
						Fiber	Fragment
Liquid soap	2	5	57	37	7	78	30
Micellar water	6	5	41	26	8	44	44
Micellar cleansing oil	1	3	45	36	14	61	39

**Table 5:** Level of MPs in the three product categories analyzed by micro-FTIR.

Samples	Cotton	Cellulose	Flax	Wool	Poly(methyl methacrylate)	Polyamide (Nylon)	Polyester	Polyethylene	Polyurethane	Other polymers
Liquid soap	52	52	8	0	12	8	2	1	0	5
Micellar water	56	17	5	4	4	3	0	2	0	7
Micellar cleansing oil	66	18	15	1	30	3	3	1	2	14
Total	174	87	28	5	46	14	5	4	2	26

**Table 6:** Pearson correlations for the three categories of samples.

Categories of Samples		Micellar Water		Liquid Soap		Micellar Cleansing Oil	
Coefficients		Fiber Type	Occurrence Source	Fiber Type	Occurrence Source	Fiber Type	Occurrence Source
Fiber type	Pearson Correlation	1	0.659 **	1	0.808 **	1	0.770 **
	Sig. (2-tailed)		0.000		0.000		0.000
	N	88	88	108	108	100	100
Occurrence source	Pearson Correlation	0.659 **	1	0.808 **	1	0.770 **	1
	Sig. (2-tailed)	0.000		0.000		0.000	
	N	88	88	108	108	100	100

**. Correlation is significant at the 0.01 level (2-tailed).

**Table 7:** Model summary ^b^.

Sample	R	R Square	Adjusted R Square	Std. Error of the Estimate	Change Statistics				
					R Square Change	F Change	df_1_	df_2_	Sig. F Change
Liquid soap	0.808 ^a^	0.653	0.649	4.040	0.653	199.041	1	106	0.000
Micellar water	0.659 ^a^	0.434	0.427	6.048	0.434	65.950	1	86	0.000
Micellar cleansing oil	0.770 ^a^	0.593	0.589	4.259	0.593	142.897	1	98	0.000

^a^. Predictors: constant, occurrence source. ^b^. Dependent Variable: fiber type.

**Table 8:** Results on the doses of ingestion, inhalation, absorption, and chronic total exposure dose to microplastics for the three product categories.

Product Category	Sample Code	*D_der_*	*D_ing_*	*D_inh_*	*D_TOTAL_*
		[n·L^−1^·day^−1^]	[n·L^−1^·day^−1^]	[n·L^−1^·day^−1^]	[n·L^−1^·day^−1^]
Liquid soap	LS1	1.25 × 10^−^^1^	7.14 × 10^−^^4^	1.05 × 10^−^^7^	1.26 × 10^−^^1^
	LS2	1.25 × 10^−^^1^	7.14 × 10^−^^4^	1.05 × 10^−^^7^	1.26 × 10^−^^1^
	LS3	2.25 × 10^−^^1^	1.29 × 10^−^^3^	1.89 × 10^−^^7^	2.26 × 10^−^^1^
	LS4	5.00 × 10^−^^2^	2.86 × 10^−^^4^	4.20 × 10^−^^8^	5.03 × 10^−^^2^
	LS5	2.50 × 10^−^^1^	1.43 × 10^−^^3^	2.10 × 10^−^^7^	2.51 × 10^−^^1^
	LS6	2.00 × 10^−^^1^	1.14 × 10^−^^3^	1.68 × 10^−^^7^	2.01 × 10^−^^1^
	LS7	2.50 × 10^−^^1^	1.43 × 10^−^^3^	2.10 × 10^−^^7^	2.51 × 10^−^^1^
	LS8	5.00 × 10^−^^2^	2.86 × 10^−^^4^	4.20 × 10^−^^8^	5.03 × 10^−^^2^
	LS9	5.00 × 10^−^^2^	2.86 × 10^−^^4^	4.20 × 10^−^^8^	5.03 × 10^−^^2^
	LS10	2.00 × 10^−^^1^	1.14 × 10^−^^3^	1.68 × 10^−^^7^	2.01 × 10^−^^1^
Micellar water	MW1	1.86 × 10^−^^2^	2.36 × 10^−^^3^	3.47 × 10^−^^7^	2.09 × 10^−^^2^
	MW2	1.69 × 10^−^^3^	2.14 × 10^−^^4^	3.15 × 10^−^^8^	1.90 × 10^−^^3^
	MW3	2.81 × 10^−^^3^	3.57 × 10^−^^4^	5.25 × 10^−^^8^	3.17 × 10^−^^3^
	MW4	6.19 × 10^−^^3^	7.86 × 10^−^^4^	1.16 × 10^−^^7^	6.97 × 10^−^^3^
	MW5	2.81 × 10^−^^3^	3.57 × 10^−^^4^	5.25 × 10^−^^8^	3.17 × 10^−^^3^
	MW6	2.25 × 10^−^^3^	2.86 × 10^−^^4^	4.20 × 10^−^^8^	2.54 × 10^−^^3^
Micellar cleansing oil	MCO1	1.25	7.14 × 10^−^^3^	1.05 × 10^−^^6^	1.26
	MCO2	7.50 × 10^−^^1^	4.29 × 10^−^^3^	6.30 × 10^−^^7^	7.54 × 10^−^^1^
	MCO3	3.75 × 10^−^^1^	2.14 × 10^−^^3^	3.15 × 10^−^^7^	3.77 × 10^−^^1^
	MCO4	1.00	5.71 × 10^−^^3^	8.40 × 10^−^^7^	1.01
	MCO5	3.75 × 10^−^^1^	2.14 × 10^−^^3^	3.15 × 10^−^^7^	3.77 × 10^−^^1^
	MCO6	6.25 × 10^−^^1^	3.57 × 10^−^^3^	5.25 × 10^−^^7^	6.29 × 10^−^^1^

## Data Availability

All data supporting reported results can be found in this article and in the Supplementary Materials.

## Supplementary Materials

The following supporting information can be downloaded at: https://www.mdpi.com/article/10.3390/toxics13050354/s1, Figure S1: Optical microscopy images of representative MPs identified in liquid soap samples; Figure S2: Optical microscopy images of representative MPs identified in micellar water samples; Figure S3: Optical microscopy images of representative MPs identified in the micellar cleansing oil samples; Table S1: Type and ingredients of analyzed cleanser products according to the labels; Table S2: MPs identification according to OPUS v.7.5 software spectra library (liquid soap samples); Table S3: Identification of MPs according to the OPUS v.7.5 software spectra library (micellar water samples); Table S4: MPs identification according to OPUS v.7.5 software spectra library (micellar cleansing oil samples).

## Abbreviations

The following abbreviations are used in this manuscript:

AT	average exposure time
ATR	attenuated total reflection
BW	body mass
CF	conversion factor
CI 14720	carmoisine
CSF	Cancer slope factor
DEA	diethanolamine
DPG	dipropylene glycol
EC	emerging contaminant
ECHA	European Chemical Agency
ED	exposure duration
EDTA	ethylene-diamine-tetra-acetic acid
EF	exposure frequency
ESR	Institute of Environmental Science and Research Limited
FDA	U.S. Food and Drug Administration
FTIR	Fourier transform infrared spectroscopy
HA	hierarchical cluster analysis
HM	heavy metal
HPLC	high performance liquid chromatography
InhR	inhalation rate
IR	infrared
IR	ingestion rate
MP	microplastic
NOC	natural and organic cosmetics
OM	optical microscopy
PAH	polycyclic aromatic hydrocarbon
PCA	pyrrolidone carboxylic acid
PCB	polychlorinated biphenyl
PCCP	personal care and cosmetic product
PEF	particle emission factor
PEG	polyethylene glycol
REACH	Council on the Registration, Evaluation, Authorization, and Restriction of Chemicals
RfD	daily reference dose
SA	exposed surface area
SC	stratum corneum
SCCS	Scientific Committee on Consumer Safety
TEA	triethanolamine
US EPA	U.S. Environmental Protection Agency
WHO	World Health Organization
