# Supplementary material for: Potential Health Risk of Microplastic Exposures from Skin-Cleansing Products
Source: Toxics. 2025 Apr 29;13(5):354. doi: 10.3390/toxics13050354 (PMC12116115; doi:10.3390/toxics13050354)
Supplement: Supplementary file 1 [file toxics-13-00354-s001.zip › toxics-3591935-supplementary.pdf]

toxics

MDPI

Supplementary Material

Potential Health Risk of Microplastic Exposures from Skin-Cleansing Products

Raluca Maria Bucur (Popa)[1,2], Cristiana Radulescu[1,3,4,*], Ioana Daniela Dulama[5,*], Raluca Maria Stirbescu[5], Ioan Alin Bucurica[5], Andreea Laura Banica[1,5], Sorina Geanina Stanescu[5]

[1] National University of Science and Technology Politehnica of Bucharest, Doctoral School Chemical Engineering and Biotechnology, 060042 Bucharest, Romania; e-mail: ralucamaria.bucur@gmail.com (R.M.B.P.)
[2] Valahia University of Targoviste, Research and Expertise Center for Natural Resources and Environment, 130004 Targoviste, Romania;
[3] Valahia University of Targoviste, Faculty of Sciences and Arts, 130004 Targoviste, Romania; e-mail: cristiana.radulescu@valahia.ro
[4] Academy of Romanian Scientists, 050044 Bucharest, Romania;
[5] Valahia University of Targoviste, Institute of Multidisciplinary Research for Science and Technology, 130004 Targoviste, Romania; e-mail: dulama.ioana@icstm.ro (I.D.D.); stirbescu.raluca@icstm.ro (R.M.S.); bucurica_alin@icstm.ro (I.A.B.); banica.andreea@icstm.ro (A.L.B.); geanina.stanescu@icstm.ro (S.G.S.);
* Corresponding author: cristiana.radulescu@valahia.ro (C.R.); dulama.ioana@icstm.ro (I.D.D.).

Supplementary Material contents:

Table S1. Type and ingredients of analyzed cleanser products according to the labels.
Figure S1. Optical microscopy images of representative MPs identified in liquid soap samples.
Figure S2. Optical microscopy images of representative MPs identified in micellar water samples
Figure S3. Optical microscopy images of representative MPs identified in the micellar cleansing oil samples.
Table S2. MPs identification according to OPUS v.7.5 software spectra library (liquid soap samples)
Table S3. Identification of MPs according to the OPUS v.7.5 software spectra library (micellar water samples)
Table S4. MPs identification according to OPUS v.7.5 software spectra library (micellar cleansing oil samples)

Table S1. Type and ingredients of analyzed cleanser products according to the labels.

Cleanser type	Code	Ingredients declared by the producer on the label
Liquid soap	LS1	Water, sodium lauryl sulfate, cocamidopropyl betaine, glycerin, fragrance, glycerin, rosemary oil, thyme oil, citric acid, fragrance, tetrasodium EDTA, methylchloroisothiazolinone, methylisothiazolinone.
	LS2	Water, sodium lauryl sulfate, sodium chloride, cocamidopropyl betaine, glycerin, cocamide DEA, polyquaternium-7, TEA-lauryl sulfate, styrene/acrylates copolymer, fragrance, methylchloroisotiazolinone, methylisothiazolinone, honey extract, citric acid, glyceric ester.
	LS3	Water, glycerin, sodium lauryl sulfate, cocamidopropyl betaine, PEG-7, PEG-40, citric acid, fragrance, sodium chloride, potassium sorbate, hexyl cinnamal.
	LS4	Sodium palmate, sodium oleate, water, glycerin, sodium laurate, fragrance, sodium chloride, linseed oil, EDTA, calendula extract, camellia extract, *Melaleuca alternifolia* leaf oil, *Rosemary* extract, *Thymus vulgaris* extract, benzyl salicylate, citral, coumarin, limonene, linalool.
	LS5	Water, glycerin, sodium chloride, sodium C14-16 olefine sulfonate, cocamidopropyl betaine, hydrolyzed milk protein, Olea Europaea (olive) fruit extract, fragrance, hydroxypropyl guar hydroxypropyl trimonium chloride, sodium benzoate, potassium sorbate, citric acid, sodium sulfate
	LS6	Water, sodium lauryl sulfate, glycerin, sodium chloride, cocamidopropyl betaine, fragrance, PEG-200, sodium benzoate, citric acid, potassium sorbate, PEG-7, sodium hydroxide, hexyl cinnamal, aloe.
	LS7	Water, sodium chloride, sodium lauryl sulfate, cocamidopropyl betaine, glycerin, perfume, PEG 55, sodium hydroxide, citric acid, PEG-55 sodium benzoate, benzyl salicylate, citric acid, hexyl cinnamal, linalool.
	LS8	Water, sodium cocoamphoacetate, cocamidopropyl betaine, isostearamide, citric acid, aloe, jasmine extract, glycerin, levulinic acid, perfume, potassium sorbate, sodium benzoate
	LS9	Water, sodium chloride, sodium lauryl sulfate, cocamidopropyl betaine, glycerin, fragrance, cocamide monoethanolamine, styrene/acrylates copolymer, sodium salicylate, sodium benzoate, polyquaternium-7, citric acid, tetrasodium EDTA, olive oil, benzyl salicylate, butylphenyl methylpropional, hexyl cinnamal, linalool.
	LS10	Water, sodium cocoamphqacetate, sodium chloride, methylpropanediol, disodium EDTA, lactic acid, zinc sulfate, copper sulfate, potassium sorbate, xylitol, mannitol, rhamnose, propylene glycol, sodium benzoate, *Ginkgo biloba* leaf extract, fragrance.
Micellar water	MW1	Water, isododecane, glycerin, panthenol, 1,2-hexanediol, caprylic/capric triglyceride, sodium chloride, sodium benzoate, disodium EDTA, citric acid, CI 14720
	MW2	Water, PEG-40 hydrogenated castor oil, *Vitis vinifera* seed oil, panthenol, glyceryl glucoside, glycerin, sorbitol, decyl glucoside, poloxamer 124, polyquaternium-10, sodium cocoyl glutamate, citric acid, sodium chloride, sodium acetate, propylene glycol, 1,2-hexanediol, trisodium EDTA, phenoxyethanol.
	MW3	Water, glycerin-7 caprylate/caprate, PEG-20 glyceryl triisostearate, sodium hyaluronate, allantoin, alpha-glucan oligosaccharide, betaine, cetrimonium bromide, citric acid, disodium EDTA, fructose, glucose, glycerin, lactobacillus ferment, maltose, panthenol, fragrance, pentylene glycol, phospholipids, sodium chloride, sodium citrate, sodium hydroxide, sodium lactate, sodium PCA, sphingolipids, spirulina platensis extract, tocopherol, trehalose, urea, chlorphenesin, p-anisic acid, phenoxyethanol, citronellol, hexyl cinnamal, linalool.
	MW4	Water, pentylene glycol, polysorbate 20, sodium citrate, citric acid, disodium phosphate, fragrance.
	MW5	Water, polyglyceryl-4 caprate, glycerin, sodium cocoyl apple amino acids, camelia sinensis leaf extract, sodium hyaluronate, phenoxyethanol, ethylhexylglycerin, tetrasodium EDTA, citric acid.

Cleanser type	Code	Ingredients declared by the producer on the label
	MW6	Water, glycerin, PEG-6 caprylic/capric glycerides, PPG-26-buteth-26, PEG-40 hydrogenated castor oil, phenoxyethanol, ethylhexylglycerin, sodium hyaluronate, superoxide dismutase, fragrance, pentylene glycol, tromethamine.
Micellar cleansing oil	MCO1	Water, glycerin, PEG-200 hydrogenated glyceryl palmate, coco-betaine, disodium cocoyl glutamate, PEG-120 methyl glucose dioleate, polysorbate 20, PEG-7 glyceryl cocoate, PEG-150 pentaerythrityl tetrastearate, PPG-5-ceteth-20, PEG-6 caprylic/capric glycerides, squalane, ceramide, carbomer, triethyl citrate, sodium chloride, sodium hydroxide, sodium cocoyl glutamate, sodium lauryl lactylate, sodium hyaluronate, cholesterol, citric acid, capryloyl glycine, hydroxy acetophenone, caprylyl glycol, caprylic/capric triglyceride, trisodium ethylenediamine disuccinate, phytosphingosine, xanthan gum, benzoic acid.
	MCO2	Helianthus annuus (sunflower) seed oil, polyglycerol-4 oleate, *Carthamus tinctorius* (safflower) seed oil, fragrance, tocopherol, linalool, limonene, citral.
	MCO3	Mineral oil, isopropyl myristate, PEG-7 glyceryl coconut oil fatty acid, dicaprylyl ether, PEG-8 glyceryl isostearate, PEG-20 glyceryl triisostearate, squalane, poly glyceryl-3 diisostearate, hydrogenated polyisobutylene, octyldodecanol, *Caninabara* fruit oil, jojoba seed oil, rosemary leaf extract, DPG, tocopherol, fragrance.
	MCO4	Water, sodium PCA, propanediol, caprylic/capric triglyceride, benzo triazolyl dodecyl p-cresol, tocopherol, *Helianthus annuus* (sunflower) seed oil, *Laminaria ochroleuca* extract, stearyl glycyrrhizinate.
	MCO5	Water, cyclopentasiloxane, isohexadecane, potassium phosphate, sodium chloride, hexylene glycol, dipotassium phosphate, disodium EDTA, decyl glucoside, polyaminopropyl biguanide, *Argania spinosa* kernel oil, limonene, linalool, ethylhexyl salicylate, butyl methoxydibenzoyl methane, pentaerythrityl tetra-di-t-butyl hydroxyhydrocinnamate, geraniol, *Haematococcus pluvialis* extract, caprylic/capric triglyceride, tocopherol, fragrance
	MCO6	Dibutyl adipate, caprylic triglycerides, diethylamino hydroxy benzoyl hexyl benzoate, homosalate, bisethylhexyloxyphenol methoxyphenyl, triazine, diisopropyl sebacate, ethylhexyl salicylate, ethylhexyl triazine, oleoyl tyrosine, *Luffa cylindrica* seed oil, oleic acid, tocopherol, glycine soybean oil, fragrance

EDTA=ethylene-diamine-tetra-acetic acid; DEA=diethanolamine; TEA=triethanolamine; PEG=polyethylene glycols; CI 14720=carmoisine; PCA=pyrrolidone carboxylic acid; PPG-5-ceteth-20=hexadecyl ether; DPG=dipropylene glycol.

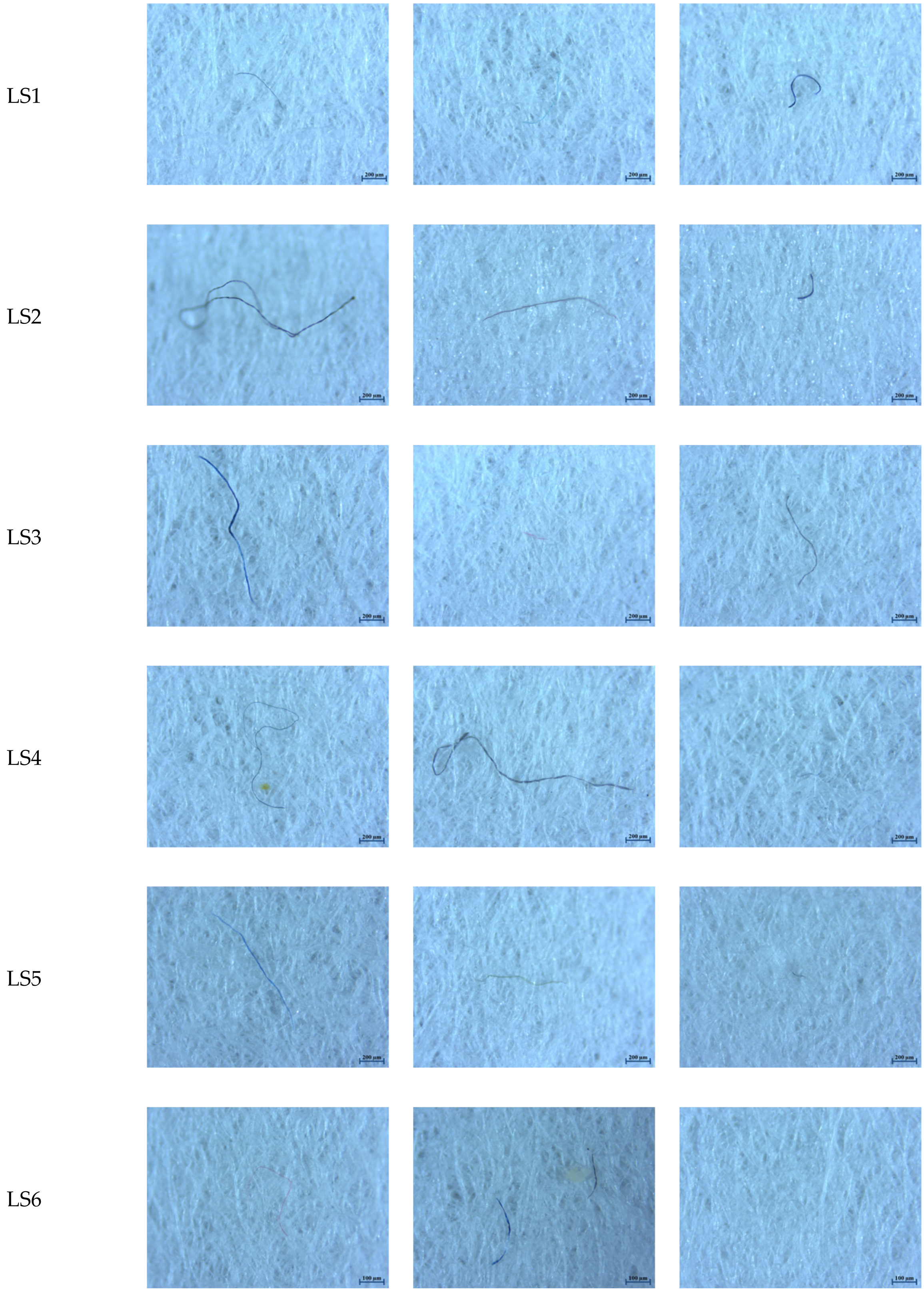
LS1
LS2
LS3
LS4
LS5
LS6

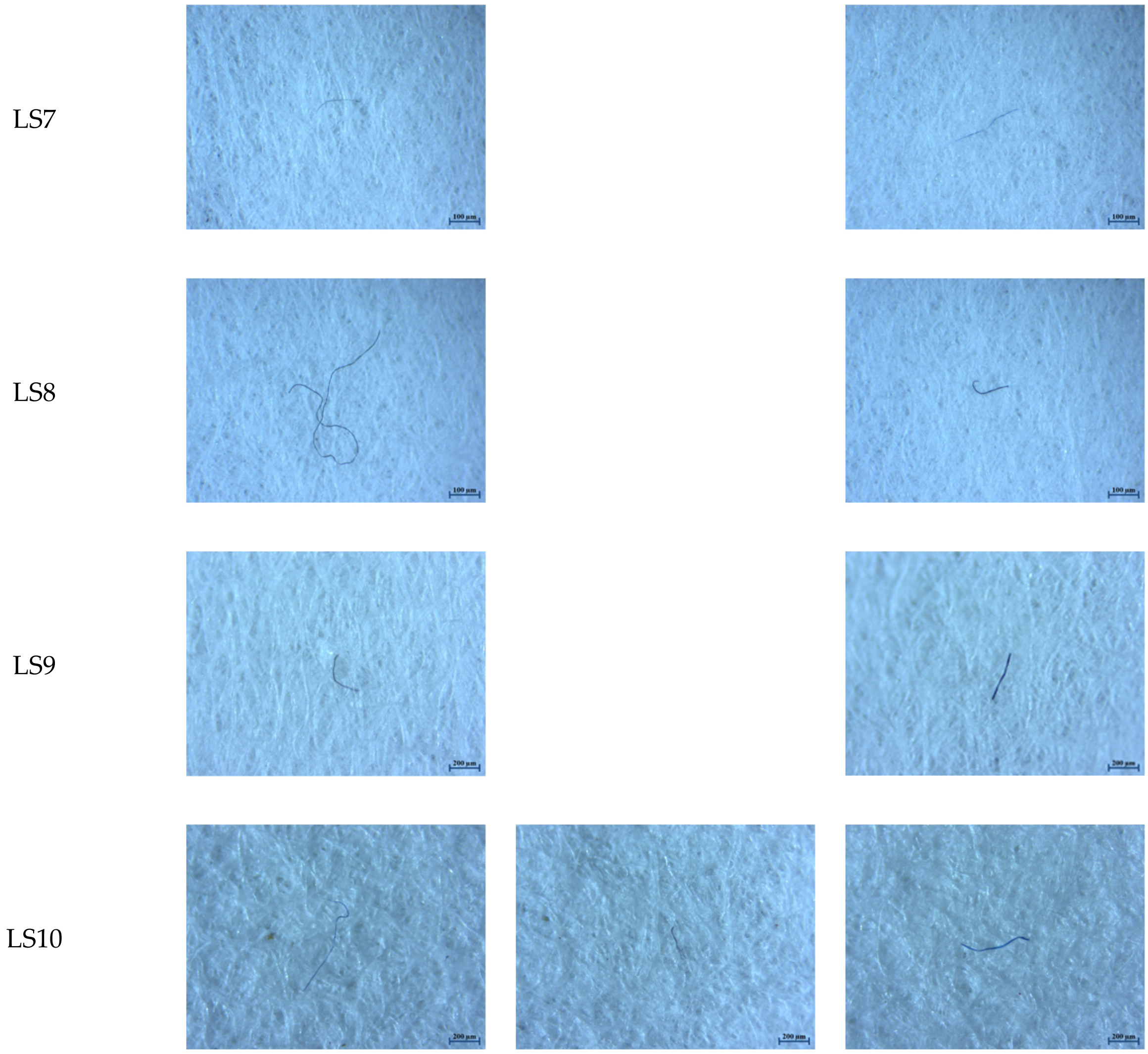

Figure S1. Optical microscopy images of representative MPs identified in liquid soap samples.

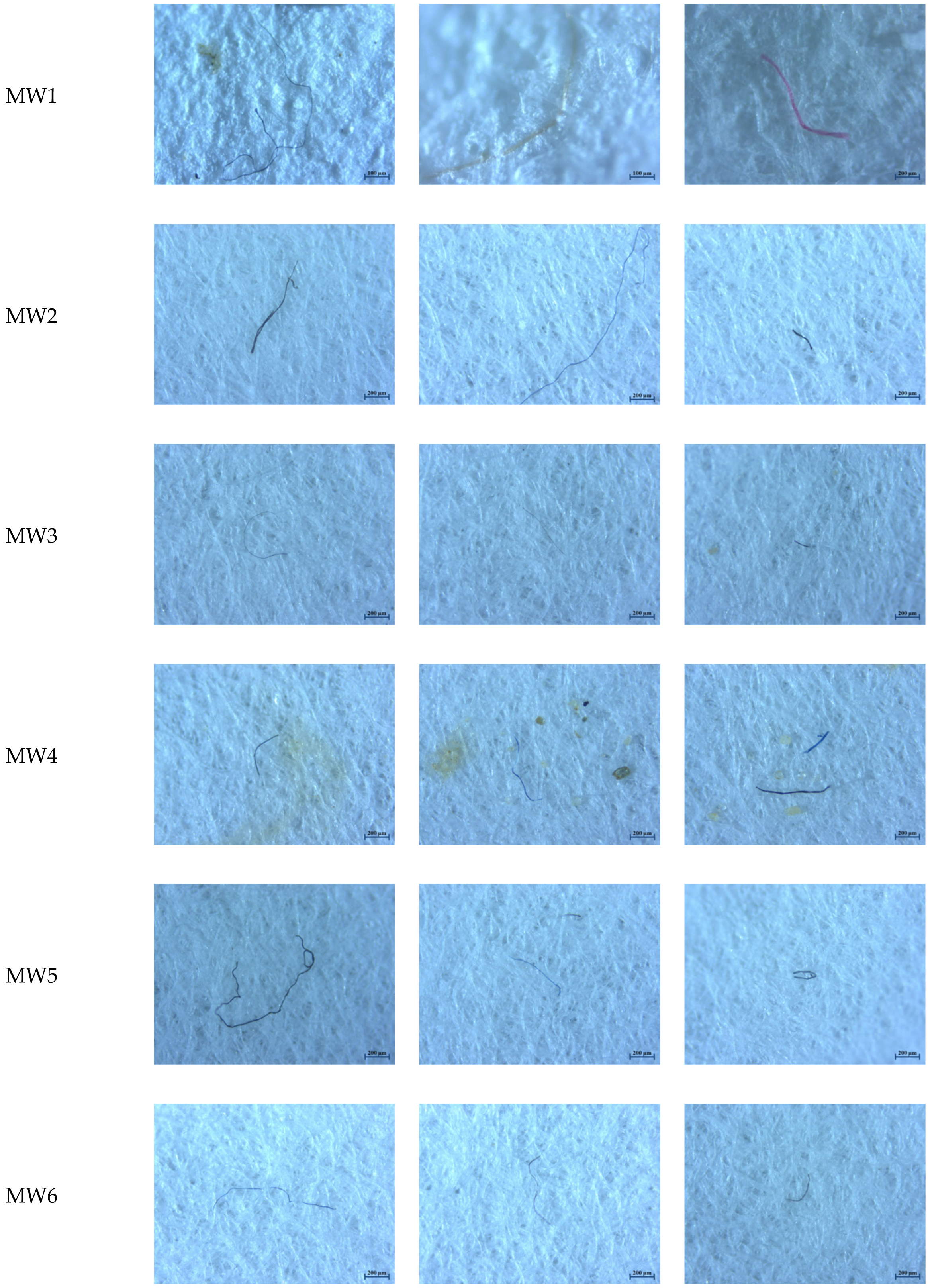

Figure S2. Optical microscopy images of representative MPs identified in micellar water samples

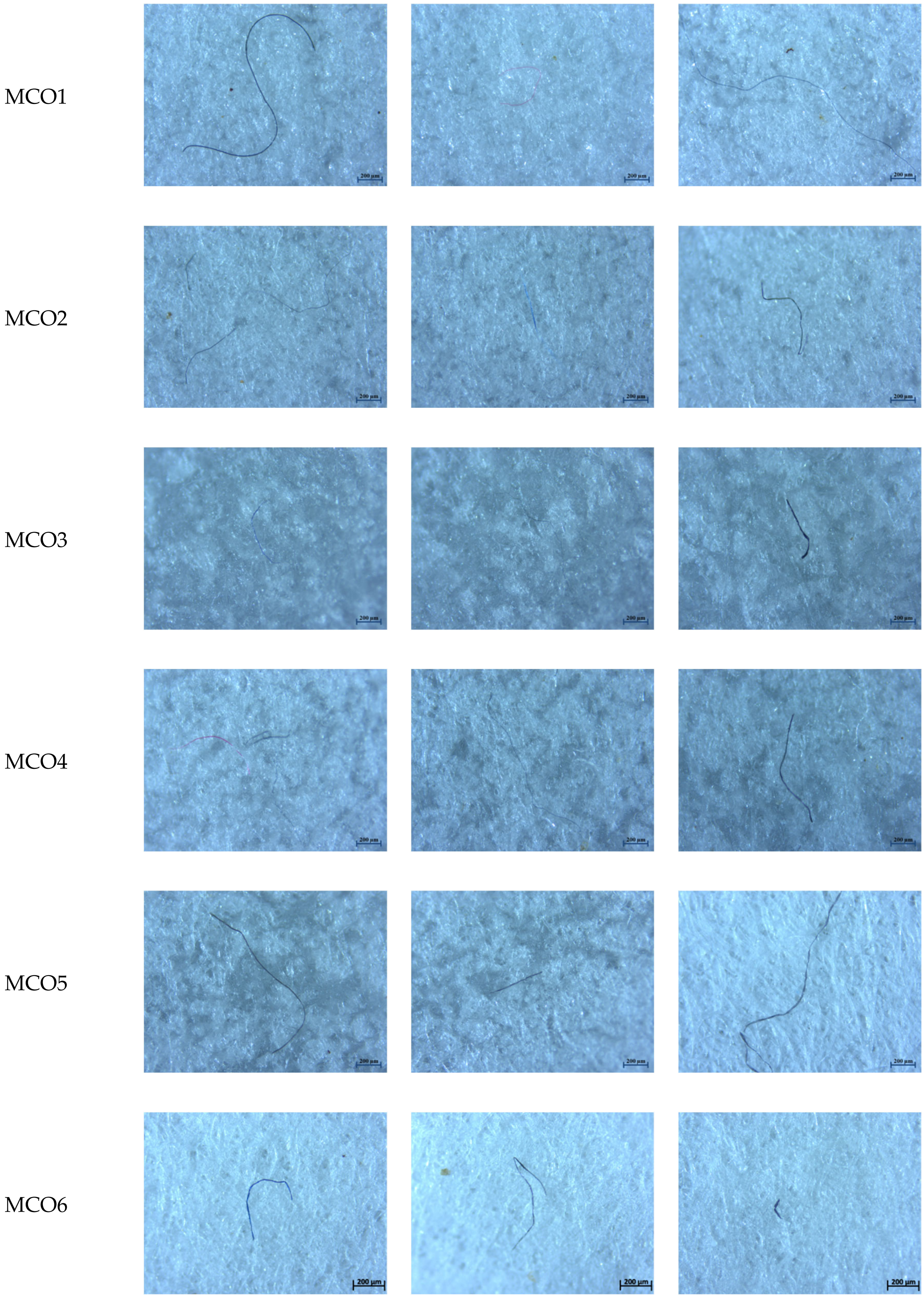

Figure S3. Optical microscopy images of representative MPs identified in the micellar cleansing oil samples.

Table S2. MPs identification according to OPUS v.7.5 software spectra library (liquid soap samples)

Sample code	Micro-FTIR image	Composition of microplastics [%]						Shape
		Cotton	Poly(methyl methacrylate)	Polyamide (Nylon)	Cellulose	Polyester	Polyethylene	
LS1.1	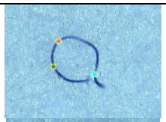	43	6	-	25	25		Fiber
LS1.2	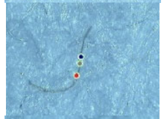	70	6	11	13	-	-	Fiber
LS1.3	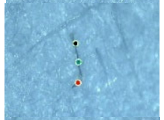	70	6	11	13	-	-	Fiber
LS1.4	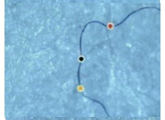	-	-	-	-	100	-	Fiber
LS2.1	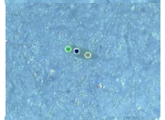	60	40	-	-	-	-	Elongated fragment
LS2.2	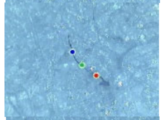	60	40	-	-	-	-	Fiber
LS2.3	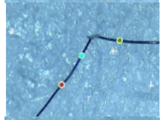	60	40	-	-	-	-	Fiber
LS2.4	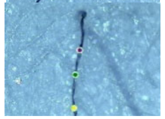	50	50	-	-	-	-	Fiber

Sample code	Micro-FTIR image	Composition of microplastics [%]						Shape
		Cotton	Poly(methyl methacrylate)	Polyamide (Nylon)	Cellulose	Polyester	Polyeth-ylene	
LS2.5	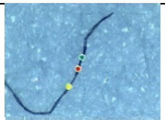	79	21	-	-	-	-	Fiber
LS3.1	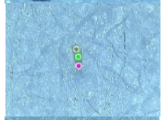	60	40	-	-	-	-	Irregular fragment
LS3.2	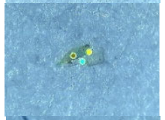	70	6	11	13	-	-	Angular fragment
LS3.3	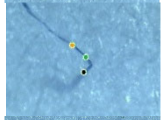	47	-	2	51	-	-	Fiber
LS5.1	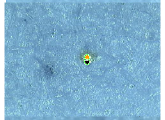	47	-	2	51	-	-	Irregular fragment
LS5.2	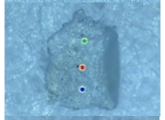	-	-	-	-	-	100	Angular fragment
LS5.3	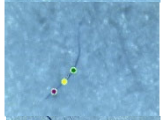	47	-	2	51	-	-	Fiber
LS5.4	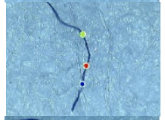	70	6	11	13	-	-	Fiber
LS7.1	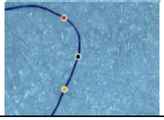	70	6	11	13	-	-	Fiber

Table S3. Identification of MPs according to the OPUS v.7.5 software spectra library (micellar water samples)

Sample code	Micro-FTIR image	Composition of microplastics [%]						Shape
		Cotton	Poly(methyl methacrylate)	Polyamide (Nylon)	Cellulose	Polyester	Polyeth-ylene	
MW2.1	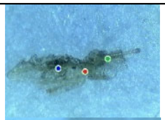	-	-	-	-	-	100	Irregular fragment
MW2.2	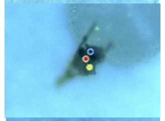	-	-	-	-	-	100	Irregular fragment
MW2.3	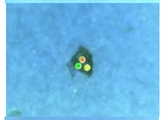	79	21	-	-	-	-	Irregular fragment
MW2.4	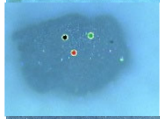	-	-	-	-	-	100	Irregular fragment
MW4.1	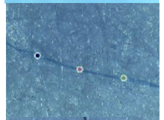	65	-	25	10	-	-	Fiber
MW4.2	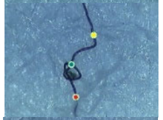		-	-	-	100	-	Fiber
MW4.3	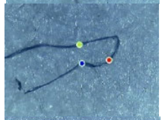	65	-	25	10	-	-	Fiber
MW5.1	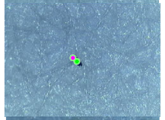	47	-	2	51	-	-	Irregular fragment
MW6.1	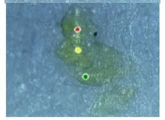	-	-	100	-	-	-	Irregular fragment

Sample code	Micro-FTIR image	Composition of microplastics [%]						Shape
		Cotton	Poly(methyl methacrylate)	Polyamide (Nylon)	Cellulose	Polyester	Polyeth-ylene	
MW6.2	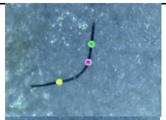	43	6	-	25	25	-	Fiber
MW7.1	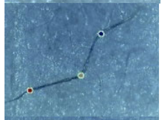	70	6	11	13	-	-	Fiber
MW7.2	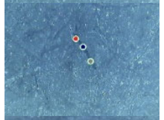	70	6	11	13	-	-	Fiber
MW7.3	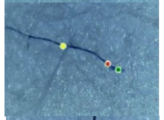	70	6	11	13	-	-	Fiber
MW7.4	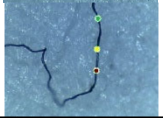	65	-	25	10	-	-	Fiber

Table S4. MPs identification according to OPUS v.7.5 software spectra library (micellar cleansing oil samples)

Sample code	Micro-FTIR image	Composition of microplastics [%]							
		Cotton	Poly(methyl methacrylate)	Polyamide (Nylon)	Polyurethane	Cellulose	Polyeth-ylene	Polyester	Shape
MCO1.1	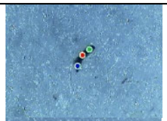	79	21	-	-	-	-	-	Elongated fragment
MCO1.2	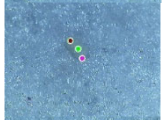	70	6	11	-	13	-	-	Elongated fragment
MCO1.3	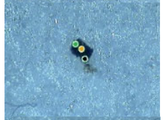	50	-	-	50	-	-	-	Angular fragment
MCO1.4	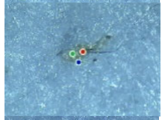	60	40	-	-	-	-	-	Irregular fragment
MCO1.5	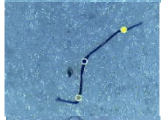	79	21	-	-	-	-	-	Fiber
MCO1.6	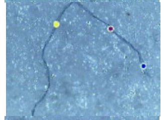	-	50	-	-	50	-	-	Fiber
MCO1.7	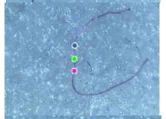	-	-	-	-	-	-	100	Fiber
MCO1.8	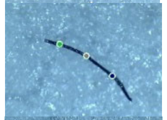	64	15	-	-	-	-	21	Fiber
MCO1.9	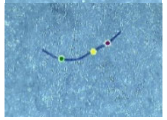	50	50	-	-	-	-	-	Fiber

Sample code	Micro-FTIR image	Composition of microplastics [%]							
		Cotton	Poly(methyl methacrylate)	Polyamide (Nylon)	Polyurethane	Cellulose	Polyeth-ylene	Polyester	Shape
MCO3.1	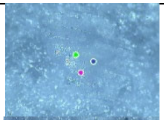	-	-	-	-	-	100	-	Irregular fragment
MCO3.2	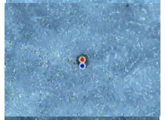	60	40	-	-	-	-	-	Irregular fragment
MCO3.3	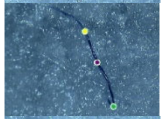	79	21	-	-	-	-	-	Fiber
MCO3.4	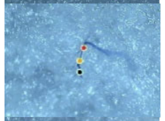	60	40	-	-	-	-	-	Fiber
MCO3.5	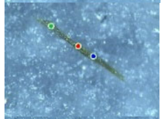	79	21	-	-	-	-	-	Fiber
MCO3.6	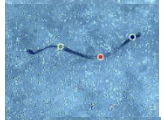	60	40	-	-	-	-	-	Fiber
MCO3.7	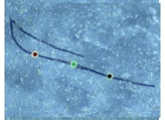	50	50	-	-	-	-	-	Fiber
MCO6.1	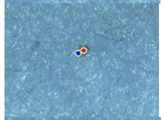	70	6	11	-	13	-	-	Irregular fragment
MCO6.2	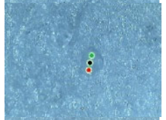	50	50	-	-	-	-	-	Irregular fragment
MCO6.3	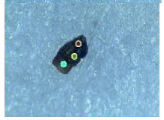	70	6	11	-	13	-	-	Angular fragment

Sample code	Micro-FTIR image	Composition of microplastics [%]							
		Cotton	Poly(methyl methacrylate)	Polyamide (Nylon)	Polyurethane	Cellulose	Polyeth-ylene	Polyester	Shape
MCO6.4	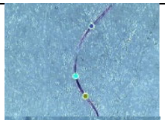	50	50	-	-	-	-	-	Fiber
MCO2.1	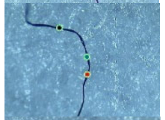	92	-	-	8	-	-	-	Fiber
MCO2.2	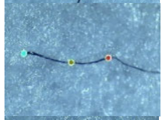	79	21	-	-	-	-	-	Fiber
MCO4.1	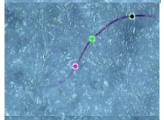	79	21	-	-	-	-	-	Fiber
MCO4.2	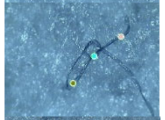	79	21	-	-	-	-	-	Fiber
MCO4.3	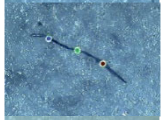	79	21	-	-	-	-	-	Fiber
MCO4.4	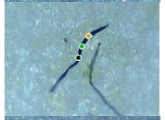	50	50	-	-	-	-	-	Fiber
MCO5.1	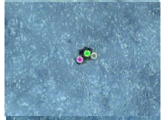	60	40	-	-	-	-	-	Angular fragment
MCO5.2	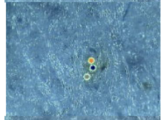	50	50	-	-	-	-	-	Irregular fragment
MCO5.3	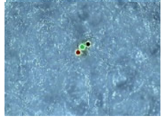	79	21	-	-	-	-	-	Irregular fragment

Sample code	Micro-FTIR image	Composition of microplastics [%]							
		Cotton	Poly(methyl methacrylate)	Polyamide (Nylon)	Polyurethane	Cellulose	Polyeth-ylene	Polyester	Shape
MCO5.4	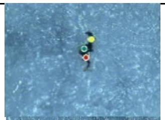	79	21	-	-	-	-	-	Elongated fragment
MCO5.5	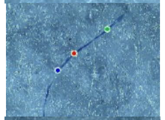	79	21	-	-	-	-	-	Fiber
MCO5.6	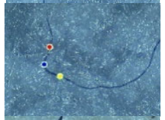	60	40	-	-	-	-	-	Fiber
MCO5.7	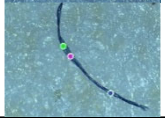	79	21	-	-	-	-	-	Fiber
